# Present and Future Surgical Options for Tricuspid Regurgitation

**DOI:** 10.31083/j.rcm2505180

**Published:** 2024-05-21

**Authors:** Ana Paula Tagliari, Maurizio Taramasso

**Affiliations:** ^1^Instituto de Ciências Biológicas e da Saúde, Universidade Federal do Rio Grande do Sul, 90046-900 Porto Alegre, Brazil; ^2^Cardiac Surgery Department, Hospital Mãe de Deus, 90880-481 Porto Alegre, Brazil; ^3^Clinic of Cardiac Surgery, HerzZentrum Hirslanden Zurich, 8008 Zurich, Switzerland

**Keywords:** tricuspid valve, tricuspid regurgitation, surgery, heart failure, right ventricle

## Abstract

Tricuspid regurgitation, once considered a relatively benign condition, has now gathered significant attention due to new evidence showing its impact on both short- and long-term follow-up. While surgical intervention remains the established standard approach for treating severe tricuspid regurgitation, current guidelines provide Class I indication for intervention in only a limited set of scenarios. This review delves into the present and future perspectives of surgical tricuspid regurgitation management, examining aspects such as disease prognosis, surgical indications, outcomes, and a comprehensive overview of past and upcoming clinical trials.

## 1. Introduction 

Tricuspid regurgitation (TR), once deemed a relatively benign condition, has now received significant attention due to its impact on both prognosis and quality of life. Consequently, dedicated congress sessions and task forces have been established to discuss TR prognosis and management. Additionally, numerous studies on new TR interventional approaches have been published.

Despite this, the role of TR surgical intervention remains limited, with only a few scenarios receiving a Class I indication for intervention according to current guidelines [1, 2]. The restricted number of patients undergoing TR surgery, especially for isolated TR, can be attributed to two main factors:

(1) The poor post-operative prognosis, which has remained consistently stable over the last few decades even with ongoing surgical improvements;

(2) The advanced disease stage in which patients with TR are referred for surgery, often in the presence of multi-organ failure.

These factors have compelled the scientific community to explore new technologies capable of addressing TR with a lower procedural risk, providing less invasive alternatives for high surgical risk patients. In this setting, transcatheter tricuspid valve intervention (TTVI) has not only provided a novel alternative for critically ill patients but has also given rise to an entirely new field of research. This includes the development of new clinical trial definitions, disease severity classification, and tailored risk scoring systems [3, 4].

This review addresses the current and future landscape of TR surgical management, focusing on disease prognosis, surgical indications, outcomes, and past and forthcoming clinical trials.

## 2. TR Classification

Since the early 1950s, a distinction between organic and functional TR has been established [5]. According to this classification, organic or primary TR (PTR) arises from primary abnormalities in the tricuspid valve (TV) apparatus in the absence of significant left-sided heart disease or pulmonary hypertension (PH) [6]. PTR can be further categorized into degenerative, congenital or acquired etiologies.

Functional or secondary TR (STR) accounts for over 85% of cases and is characterized by tricuspid annular (TA) dilatation and/or leaflet tethering in the setting of right ventricle (RV) remodeling due to pressure and/or volume overload [7, 8] with left-sided heart disease and/or PH being the most prevalent etiologies [9, 10]. A subgroup of patients presents isolated TR due to TA dilation probably attributed to atrial fibrillation (AF) [1].

Besides this standard classification, the Tricuspid Valve Academic Research Consortium (TVARC) document suggested dividing STR into three subcategories as presented in Table 1 (Ref. [11]).

**Table 1.** **Suggested STR classification according to TVARC document**.

Causative Disease Process		Etiology	TV/RV Morphology
Primary TR (5%–10%)			
	Degenerative disease	Prolapse or flail leaflet	Abnormal leaflet mobility, normal RV
	Congenital	Apical displacement of leaflet attachment (i.e., Ebstein’s anomaly)	Abnormal leaflet position, atrialized RV
	Acquired (i.e., tumors, trauma, carcinoid, RHD, radiation)	Leaflet injury (i.e., tumor, trauma, biopsy, lead extraction) or infiltration/fibrosis (i.e., carcinoid, rheumatic disease, radiation valvulopathy)	Abnormal leaflet morphology/mobility, normal RV
Secondary TR (80%)			
Ventricular secondary TR			
	LV disease	Postcapillary PH (HFpEF, HFrEF)	RV dilatation (spherical remodeling)/dysf- unctional → leaflet tethering, dilated RA/TA
	Left heart valvular disease	Postcapillary PH	
	Pulmonary disease	Pre-capillary PH (chronic lung disease, CTEPH, PAH)	
	RV dysfunction/remodeling	RV dilatation and dysfunction (i,e., RV infarct, RV dysplasia)	
Atrial secondary TR			
	RA/TA dilatation	RA/TA dilatation (i.e., related to age, AF, HFpEF)	RA dilatation/dysfunction → TA dilatation (minimal leaflet tethering), conical RV remodeling
CIED-related TR (10%–15%)			
	LTR-A (causative)	Leaflet impingement, perforation, valvular/subvalvular adhesions/restriction	Tricuspid leaflet tethering/adhesions
	LTR-B (incidental)	CIED present without TV apparatus interference	Morphology dependent on primary disease process

CTEPH, Chronic thromboembolic pulmonary hypertension; HFpEF, heart failure with preserved ejection fraction; HFrEF, heart failure with reduced ejection fraction; LTR-A, lead-associated tricuspid regurgitation type A; LTR-B, lead-associated tricuspid regurgitation Type B; LV, left ventricle; PAH, pulmonary arterial hypertension; PH, pulmonary hypertension; RHD, rheumatic heart disease; RA, right atrial; RV, right ventricular; TA, tricuspid annular; TR, tricuspid regurgitation; STR, Functional or secondary TR; TVARC, Tricuspid Valve Academic Research Consortium; TV, tricuspid valve; AF, atrial fibrillation; CIED, cardiac implantable eletronic device. Adapted from Hahn *et al*. [11]

## 3. TR Incidence

A community-based study by Topilsky *et al*. [12] revealed that significant (at least moderate) TR is present in 0.55% of the population, with a higher prevalence in the female sex. TR prevalence significantly increased with age, reaching approximately 4% in patients over 75 years old [12]. These findings reinforced previous data from the Framingham Heart Study, which demonstrated that for ≥moderate TR, the prevalence varied from 1.5% in men aged 70 years or older to 5.6% in women of the same age group. According to this study, the determinants of TR were age (odds ratio [OR] 1.5/9.9 years, 95% confidence interval [CI] 1.3–1.7), body mass index (OR 0.7/4.3 kg/$m^{2}$; 95% CI 0.6–0.8), and female gender (OR 1.2, 95% CI 1.0–1.6) [13]. Additional independent predictors of TR progression are heart failure (HF), pacemaker leads, AF, and signs of left heart disease (left atrial [LA] enlargement, elevated pulmonary artery pressure [PAP], and left-sided valvular disease) [14].

In patients with degenerative mitral regurgitation (MR), the prevalence of hemodynamically significant TR was reported to be around 30% at the time of mitral valve (MV) surgery. Additionally, up to a third of patients with significant mitral stenosis exhibit TR. Nonetheless, up to 40% of patients undergoing MV surgery develop significant TR late after surgery. The pre-existence of TA dilation (diameter ≥40 mm or 21 mm/$m^{2}$ on preoperative transthoracic echocardiography), indicating a more advanced disease stage, has been proposed as a predictor of TR progression [15, 16]. Other risk factors for TR progression are the magnitude of RV dysfunction, leaflet tethering, PH, AF, or transvalvular leads [17, 18, 19].

## 4. TR Clinical Prognosis

Severe TR is associated with a dismal prognosis, leading to progressive RV dysfunction, renal and liver failure, chronic right HF, and the need for increasing doses of diuretics [11].

It has been suggested that the clinical impact of TR is directly proportional to its degree, with moderate/severe TR associated with a 2-fold increase in mortality compared to no/mild TR, irrespective of pulmonary pressures and right HF [20]. In a retrospective study involving 5223 patients, Nath *et al*. [6] demonstrated that ≥moderate TR is associated with increased mortality, irrespective of LV ejection fraction (LVEF) (hazard ratio [HR] 1.49, 95% CI 1.34–1.66 for ejection fraction (EF) <50%; HR 1.54, 95% CI 1.37–1.71 for EF ≥50%) or pulmonary artery systolic pressure (PASP) (HR 1.31, 95% CI 1.16–1.49 for PASP >40 mmHg; HR 1.32, 95% CI 1.05–1.62 for PASP ≤40 mmHg). The one-year survival rates were 91.7% with no TR, 90.3% with mild TR, 78.9% with moderate TR, and 63.9% with severe TR. Univariate analysis revealed an association between TR, RV dilation, reduced RV function, LVEF, PAP, and inferior vena cava dilation with higher mortality. Failure to promptly refer the patient for surgery was identified as the main reason for elevated surgical morbidity and mortality [6].

Similarly, in another retrospective analysis, individuals with moderate and severe TR exhibited a 2.0- to 3.2-fold increased risk of all-cause long-term mortality, even after adjusting for age and sex, compared to those with no/trivial TR (*p* < 0.001 for both comparisons). Notably, in fully adjusted models, accounting for factors such as RV systolic pressure, AF, and significant left heart disease, even individuals with mild TR faced a significantly high mortality risk (mild TR: HR 1.24; 95% CI 1.23–1.26; moderate TR: HR 1.72; 95% CI, 1.68–1.75; severe TR: HR 2.65; 95% CI, 2.57–2.73) compared to no/trivial TR [21].

From a clinical standpoint, TR patients often exhibit progressive signs of right HF, such as peripheral edema, fatigue, exercise intolerance, weight gain, hepatic dysfunction, ascites, and cardiac cachexia, irrespective of the underlying condition [22].

## 5. TR Surgical Indication 

Identifying predictors of outcomes and discriminating patients who are responders or non-responders to TR intervention is of paramount importance in guiding the decision-making process for TR surgical management [23].

**Fig. 1.**
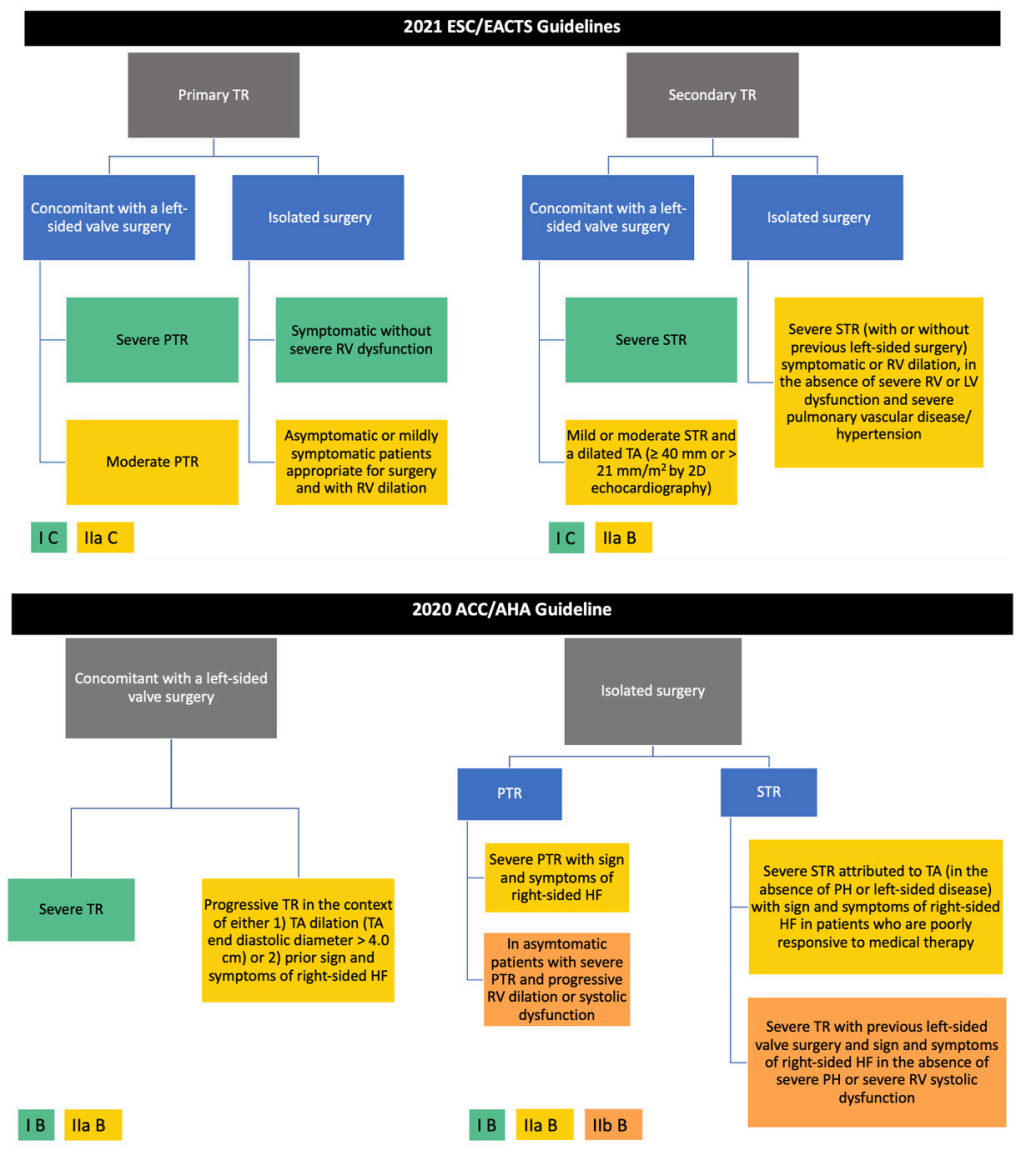
**Current indication for TR surgical intervention according to European and American guidelines [1, 2]**. ESC, European Society of Cardiology; EACTS, European Association for Cardiothoracic Surgery; ACC/AHA, American College of Cardiology/American Heart Association; TR, tricuspid regurgitation; PTR, primary tricuspid regurgitation; RV, right ventricle; LV, left ventricle; STR, secondary tricuspid regurgitation; TA, tricuspid annular.

Indications for TR surgical intervention, according to current American and European guidelines, are presented in Fig. 1 (Ref. [1, 2]). Selected patients should receive a TV repair at the time of the left-sided valve lesions surgery to address severe TR or to prevent later severe TR development in the presence of progressive TR. The rationale behind this recommendation is the understanding that severe TR may not reliably improve after left-sided lesion treatment and RV afterload reduction. In this context, a combined intervention would not increase the operative risk and could promote RV reverse remodeling and improved functional status, especially in the presence of TA dilatation. As an isolated procedure, TV surgery should be considered for selected patients with PTR or STR attributed to TA dilation, in the absence of PH or dilated cardiomyopathy. For PTR, surgery is recommended for symptomatic patients with severe regurgitation. In selected asymptomatic or mildly symptomatic patients deemed suitable for surgery, intervention should also be contemplated when RV dilatation or declining RV function is observed [1, 2].

TV reoperation for new-onset or worsening STR after left-sided surgery carries a high procedural risk, possibly due to late referral and subsequent poor clinical condition. The perioperative mortality rate for reoperation in the presence of severe, isolated TR after left-sided valve surgery is reported to be between 10% and 25% [1]. The surgical treatment should be considered if there are signs of RV dilatation or decline in RV function, after excluding left-sided valve dysfunction, severe RV or LV dysfunction, and severe pulmonary vascular disease/PH [1].

Although this article primarily delves into the surgical management of acquired TR, it is worth mentioning that in cases of congenitally dysplastic TVs, the Cone’s reconstruction technique, as described by da Silva *et al*. [24], stands as the standard approach for treating both pediatric patients and adults with Ebstein’s anomaly.

## 6. TR Surgical Outcomes

Severe isolated TR surgery historically carries a high mortality rate, ranging from 8% to 20% [25]. To improve these numbers and avoid operating on patients in a late disease stage, there has been a renewed interest in earlier surgery for patients with severe isolated TR before the onset of severe RV dysfunction or end-organ damage.

**Fig. 2.**
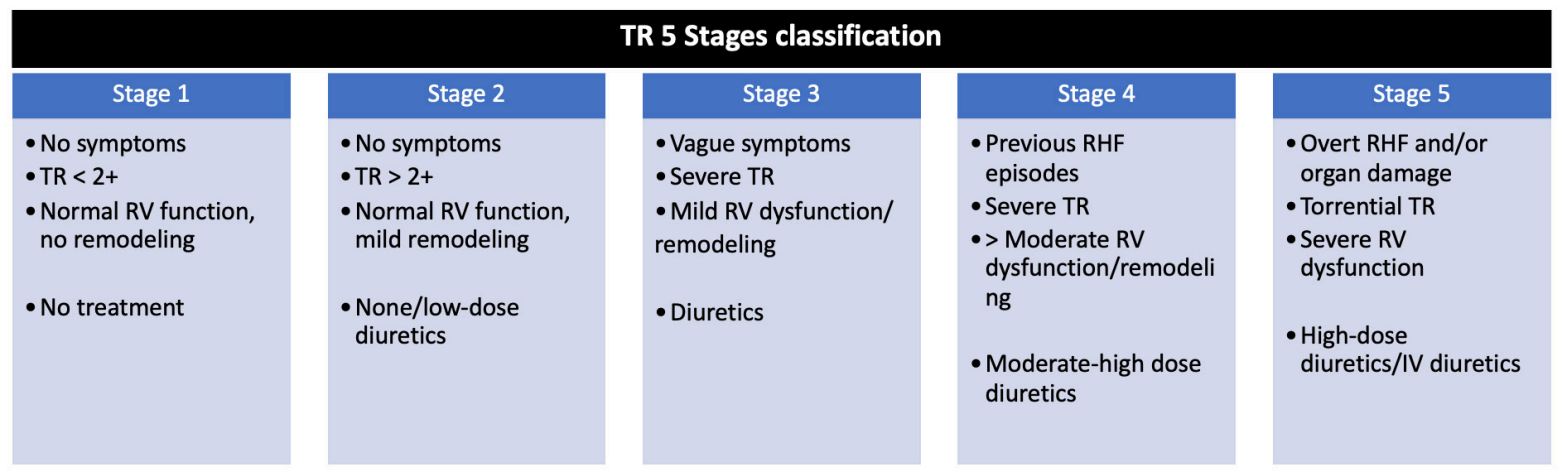
**TR 5 stages classification**. Adapted from Sala *et al*. [26]. IV, intravenous; RHF, right heart failure; RV, right ventricle; TR, tricuspid regurgitation.

According to Sala *et al*. [26], patients who underwent isolated TV surgery in early disease stages (Stages 2 and 3, see Fig. 2 (Ref. [26])), without prominent symptomatology, RV dilation or dysfunction, and organ involvement, were more likely to receive TV repair than replacement. They exhibited lower in-hospital mortality, fewer postoperative complications, shorter postoperative lengths-of-stay, and also experienced a 100% 5-year survival with no further HF rehospitalizations. Conversely, patients in advanced disease stages (Stages 4 and 5) had higher in-hospital mortality (15.3%), higher postoperative complications rate (acute kidney injury: 3.7–10% *vs.* 44–100%, *p* < 0.001; low cardiac output syndrome: 15–50% *vs.* 71–100%, *p* < 0.001), and longer intensive care unit and hospital lengths-of-stay. Their 5-year survival rate was 60.5%, with a 20% rate of right HF rehospitalization [26, 27]. Based on these data, the authors suggested that patients treated in advanced disease stages may not benefit from a reduction in venous congestion and reverse remodeling. Conversely, patients who remain symptomatic and have fluid overload despite diuretic treatment, alongside mild or moderate LV impairment, preserved RV function, no evidence of pre-capillary PH, and only mild/moderate renal and liver dysfunction, are the ones who can benefit most from TR intervention [28].

In the context of STR, Dreyfus *et al*. [15] proposed a comprehensive approach considering not only TR severity but also TA dilation, the mode of tricuspid leaflet coaptation, and tricuspid leaflet tethering. Recommendations for intervention would vary according to the disease stage. In Stage 1 (no or mild TR, TA 40 mm, and normal leaflet coaptation), TR intervention would not be indicated. In Stage 2 (mild or moderate TR, TA >40 mm, and impaired leaflet coaptation), concomitant TV annuloplasty at the time of left-sided valve disease surgery would be recommended. In Stage 3 (severe TR, TA >40 mm, impaired leaflet coaptation, and leaflet tethering with the coaptation point occurring ≥8 mm below the TA level), concomitant TV annuloplasty would be also recommended. In the presence of significant leaflet tethering, the authors suggested the anterior leaflet augmenting technique to ensure adequate long-term results and to avoid recurrent TR [15].

To better evaluate the role of concomitant TR intervention based on TA dilation, a sequence of randomized clinical trials (RCT) were conducted (Table 2, Ref. [19, 29, 30, 31]). In the first trial, Benedetto *et al*. [19] showed that patients with ≤moderate TR and TA dilatation (≥40 mm) who underwent MV surgery combined with TV annuloplasty presented less TR at 1-year follow-up, better RV reverse remodeling, and markedly improved 6-minute walk test results compared to those undergoing isolated MV surgery. Some years later, Song *et al*. [29] evaluated the results of MV surgery with or without combined TV intervention in patients with mild TR. After a 2-year follow-up, TA dimensions were significantly lower and RV fractional area change (FAC), RV ejection fraction, TR degree and 2-year survival were significantly better in those patients who received a combined procedure [29].

**Table 2.** **A summary of RCTs comparing MV surgery isolated or combined with TV annuloplasty for less than severe TR**.

Study	Publication year	Number of patients	Patient population	Primary endpoint	Follow-up	Main results
Benedetto *et al*. [19], RCT	2012	44 patients (22 concomitant intervention *vs.* 22 control group)	MV surgery indication with ≤moderate TR and TA dilatation (≥40 mm)	Moderate to severe (≥3+) STR	1-year	New onset of moderate to severe STR: 0% *vs.* 28%, *p* = 0.02;
						TR absent: 71% *vs.* 19%,* p* = 0.001;
						6-minute walk test: +115 ± 23 m distance *vs.* +75 ± 35 m distance from baseline, *p* = 0.008;
						30-day mortality: 4.4% *vs.* 4.4%
Song *et al*. [29], RCT	2016	100 patients (50 concomitant intervention *vs.* 50 control group)	MV replacement indication with mild TR	TR degree;	2-year	TR absent: 35 *vs.* 20 cases; Mild TR: 13 *vs.* 21 cases; Mild-to-moderate TR: 2 *vs.* 3 cases;
				Survival		Moderate TR: 0 *vs.* 6 cases, *p* < 0.05;
						Survival rate: 97.0% *vs.* 85.6%, *p* < 0.05
Pettinari *et al*. [30], Single-center RCT	2019	106 patients (53 concomitant intervention *vs.* 53 control group)	MV surgery indication and less-than severe STR (vena contracta <7 mm)	Freedom from ≥moderate TR;	5-year	Freedom from ≥moderate TR: 100% *vs.* 76%, *p* < 0.01;
				Freedom from severe TR;		Freedom from severe TR: 100% *vs.* 87.4%, *p* < 0.001;
				TR progression (increase >3 mm in vena contracta)		TR progression: 0% *vs.* 17.6%, *p* < 0.01;
						Freedom from cardiac-related mortality: 94.1% *vs.* 89.7%, *p* = 0.9
Gammie *et al*. [31], Multicentre RCT	2022	401 patients	Degenerative severe MR with moderate or less-than-moderate TR and TA dilatation (≥40 mm or 21 mm/m^2^)	TR reoperation, TR progression by 2 grades from baseline or the presence of severe TR, or death	2-year	Combined endpoint: 3.9% *vs.* 10.2%, *p* = 0.02;
		(198 concomitant intervention *vs.* 203 control group)				Mortality: 3.2% *vs.* 4.5%;
						TR progression: 0.6% *vs.* 6.1%

RCT, randomized clinical trial; MV, mitral valve; TV, tricuspid valve; TR, tricuspid regurgitation; TA, tricuspid annular; STR, secondary tricuspid regurgitation.

Despite these initial promising results, Pettinari *et al*. [30] suggested that in patients with less-than severe STR submitted to a TV repair at the time of MV surgery, long-term TR recurrence occurred irrespective of baseline TA dilation. Several echo parameters such as functional capacity, RV ejection fraction, RV end-systolic volume, and RV end-diastolic volume also remained similar in patients who underwent or not have a TV repair [29]. In this same line, another RCT conducted by The Cardiothoracic Surgical Trials Network (CTSN) investigators showed that in patients with severe degenerative MR and moderate or less-than-moderate TR with TA dilatation, the primary composite endpoint occurred almost exclusively in patients with moderate TR at baseline and not in those with less-than-moderate TR and TA dilatation. Nevertheless, concomitant TV surgery increased cardiopulmonary bypass time by an average of 34 minutes and resulted in a high permanent pacemaker implantation rate (14.1%) due to iatrogenic atrioventricular block [31].

In an attempt to better understand the impact of leaving ≤moderate STR untreated, Bertrand *et al*. [32] evaluated 492 patients who underwent surgery due to moderate or severe ischemic MR. In this analysis, concomitant TV surgery was performed in less than 8% of patients. Among the 2-year survivors, TR progression occurred in 6%, and 11% had ≥moderate TR. Once again, the baseline TA diameter was not predictive of TR progression (area under the curve (AUC) ≤0.65) [32].

Following these trials, two meta-analysis showed that in patients with ≤moderate TR, concomitant TV repair at the time of MV surgery had no impact on perioperative (pooled OR 0.54; 95% CI 0.25–1.15) or postoperative mortality (pooled OR 0.54; 95% CI 0.25–1.15), but resulted in a notable reduction in TR progression (pooled OR, 0.06; 95% CI 0.02–0.24) [33] and significant late onset of TR (≥moderate TR: RR 0.28, 95% CI 0.17–0.47; severe TR: RR 0.38, 95% CI 0.17–0.84) [34].

## 7. Surgical Techniques

TV surgical technique should be tailored to individual patient characteristics, disease stage, and anatomical considerations.

For STR treatment, Kay *et al*. [35] introduced a repair technique in 1965 using a 1–0 silk suture placed through the posterior leaflet, resulting in this leaflet exclusion. This technique, known as ‘bicuspidization’, had a high TR recurrence as it did not address the tendency of the anterior annulus to dilate. Seeking to stabilize the TA, De Vega proposed a suture semicircular annuloplasty technique, aiming to reduce the amount of intracardiac prosthetic material, enhance annular flexibility, and minimize the risk of conduction system injury [36]. Some years later, Carpentier introduced the concept of a prosthetic ring to reinforce the TA [37]. Annuloplasty rings offer several technical advantages over suture annuloplasty, including better tension distribution in the suture line, more standardized annular reduction, and the ability to differentially plicate an asymmetrically dilated annulus. Moreover, ring annuloplasty is easier to master and more reproducible, resulting in less residual or recurrent TR. The ring’s size is generally chosen by measuring the distance from the anteroseptal to the posteroseptal commissures and is implanted starting posteriorly (at the midpoint of the septal leaflet) and, then, proceeding counterclockwise.

Currently, three main devices are employed during TV annuloplasty: standard rigid rings, which were predominant in the 1990s; flexible bands, increasingly employed from the early 2000s; and 3-dimensional (3D) rigid rings in recent years [38]. Flexible bands allow for the natural physiological motion of the TA throughout the cardiac cycle, offering improved flexibility, a simpler design and implantation technique, and lower risks of device breakages and tricuspid stenosis. They also better preserve RV function and assist in RV functional recovery after surgery [39, 40, 41]. In contrast, 3D rings are designed to accommodate the saddle-shaped TV annulus. Another technique that may be applied in case of severe TA dilatation associated with leaflet tethering is anterior leaflet pericardial patch augmentation [42].

**Fig. 3.**
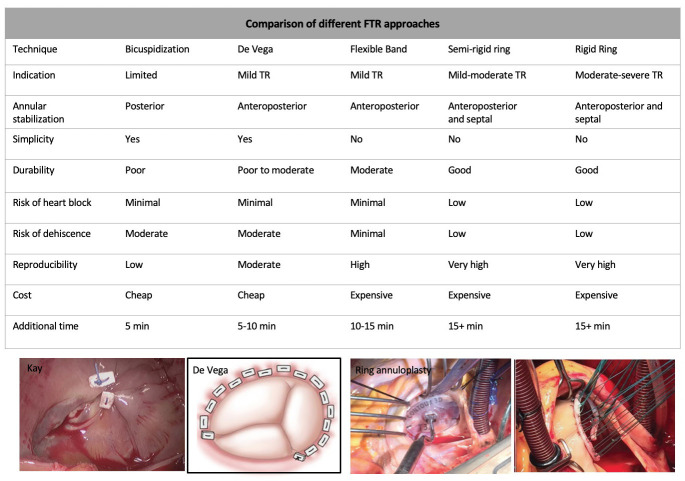
**Different approaches to treat STR**. Modified from Chikwe *et al*. [43, 44]. STR, Secondary tricuspid regurgitation; FTR, functional tricuspid regurgitation; TR, tricuspid regurgitation.

**Fig. 4.**
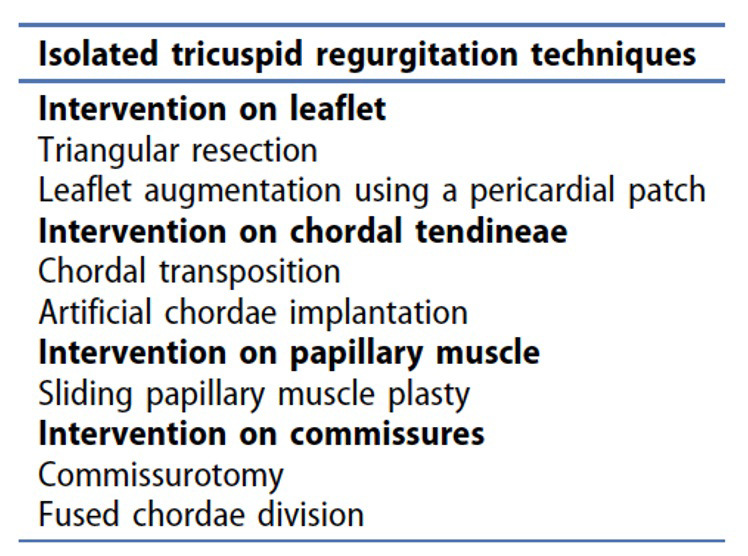
**Additional techniques for the treatment of isolated TR, which include intervention on the different components of the tricuspid valve, including the leaflets, chordal tendineae, papillary muscle and the commissures**. Modified from Belluschi *et al*. [45]. TR, tricuspid regurgitation.

Fig. 3 (Ref. [43, 44]) and Fig. 4 (Ref. [45]) show different techniques used to surgically repair STR [43, 44] or isolated TR [45].

## 8. Comparison of Different Tricuspid Valve Annuloplasty Techniques

In 1985 Rivera *et al*. [46] conducted the first RCT comparing the Carpentier tricuspid annuloplasty with the De Vega technique in 159 patients with moderate to severe TR. Over an average follow-up of 64 months, the ring annuloplasty group exhibited a significantly lower incidence of moderate or severe TR (14/41 De Vega *vs.* 4/40 Carpentier, *p* < 0.01) [46]. Subsequently, a study from Tang *et al*. [47] solidified the ring annuloplasty as the preferable approach after showing that this technique was associated with significantly better long-term survival, event-free survival, and freedom from recurrent TR in comparison to suture annuloplasty. In a multivariable analysis, ring annuloplasty was also considered an independent predictor of long-term survival (HR 0.7, 95% CI 0.5–1.0) and event-free survival (HR 0.8, CI 0.6–1.0) [47].

Regarding the best annuloplasty device, an RCT compared rigid rings versus flexible bands in 380 patients who underwent MV surgery concomitant with TV repair for STR. No difference was found in freedom from recurrent TR (97.3% in rigid ring *vs.* 96.2% in flexible band, *p* = 0.261), early mortality, overall survival, and freedom from TV reoperation. Notably, the flexible band demonstrated an advantage in restoring regional RV function, as evidenced by Doppler-derived systolic velocities of the annulus (S) and TA plane systolic excursion (TAPSE) at a 12-month follow-up [48].

Two meta-analyses also evaluated the TV annuloplasties technique. In the first, 3141 patients (1893 flexible band *vs.* 1248 rigid ring) were enrolled. There was no difference in in-hospital mortality (6.9% flexible band *vs.* 7.3% rigid ring), stroke (1.7% flexible band *vs.* 1.3% rigid rings), reoperation (*p* = 0.232), and survival (*p* = 0.086). On the other hand, the rigid ring had significantly better freedom from grade ≥2 TR at 5 years (OR 0.44; 95% CI 0.20–0.99) [49]. In the second, which included 6138 patients enrolled in suture, ring or flexible band annuloplasty, there were no significant differences in perioperative and all-cause mortality. The rigid ring group had a lower TR recurrence compared with suture annuloplasty (HR 0.42; 95% CI 0.23–0.78), while no significant difference was observed between flexible band and suture, or flexible band and rigid ring [50].

## 9. TV Replacement

Whenever possible, TV annuloplasty is preferable to valve replacement, which should only be considered when there is extensive leaflet destruction, severe tethering of TV leaflets, and significant TA dilation. When cardiac implantable electronic device leads interfere with the TV, the surgical technique should be adapted based on the patient’s condition and the surgeon’s experience [2].

In cases where replacement is indicated, a biological prosthesis is typically preferred over a mechanical one, as mechanical valves are more prone to thrombosis due to lower pressure and flow rate across the TV [51, 52]. For this same reason, the durability of a bioprosthesis in the TV position seems to be superior compared to the durability in the MV or aortic valve position. Additionally, with the emergence of TTVI, a bioprosthesis may offer the option for a future tricuspid transcatheter valve-in-valve therapy [53].

Regarding outcomes, a study by Zack *et al*. [25], which evaluated national trends and outcomes of isolated TV surgery in the United States, found that TV replacement was associated with a higher 30-day mortality rate (OR 1.91, 95% CI 1.18–3.08), an increased blood transfusion rate (39.3% *vs.* 33.2%, *p* < 0.001), and a higher need for permanent pacemaker implantation (35.0% *vs.* 13.4%, *p* < 0.001) compared with TV repair [25].

## 10. Minimally Invasive Tricuspid Valve Surgery (MIC-TVS)

TV surgery through a right mini-thoracotomy, as opposed to conventional sternotomy, has demonstrated favorable midterm outcomes. This approach is associated with reduced wound infection, lower bleeding, less pain, and a quicker return to normal life [54, 55]. Right mini-thoracotomy can be used for combined MV and TV intervention, yielding a 5-year estimated survival of 81.3%, and a 5-year freedom from reoperation rate of 100% [56]. It can also be used in patients with previous cardiac surgery with a 5-year survival rate of 72.2% [57].

Beyond minimally invasive access, isolated TV repair can also be performed through a beating heart procedure. In a multicenter study, TV-beating heart surgery was associated with a lower rate of acute renal failures and stroke compared with the arrested heart strategy. Patients undergoing a beating heart approach presented a 30-day mortality of 5%; with a 6-year survival and freedom from cardiac death of 78% ± 5% and 84% ± 4%, respectively. The 6-year composite cardiac endpoint rate, including cardiac death and reoperation, was found to be worse in the arrested heart TV surgery group than in the TV-beating heart surgery group (*p* = 0.024) [58].

## 11. TRI-SCORE

**Fig. 5.**
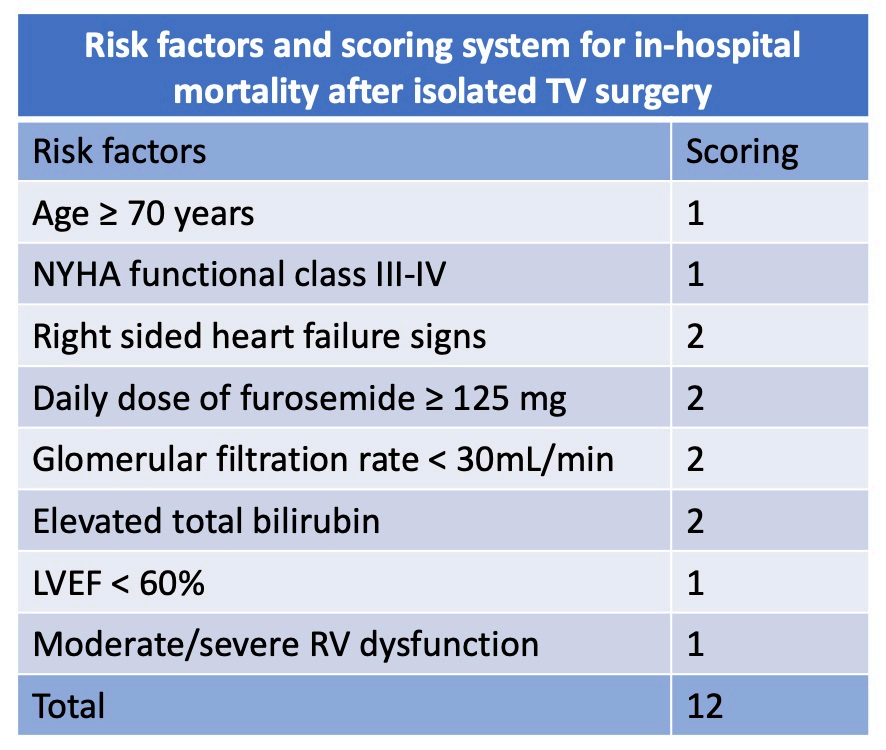
**TRI-SCORE variables. Adapted from Dreyfus *et al*. [59]**. LVEF, left ventricle ejection fraction; NYHA, New York Heart Association; RV, right ventricle; TV, Tricuspid valve.

Considering that both the Society of Thoracic Surgeons (STS) and logistic EuroSCORE/EuroSCORE II were not proposed to predict TV intervention outcomes, the TRI score was developed as a dedicated TV risk score model. The TRI-SCORE was validated in a study based on a large consecutive cohort of 466 patients who underwent isolated TV surgery for severe TR at 12 French tertiary centers. The final risk score ranged from 0 to 12 points and incorporated 8 parameters, as shown in Fig. 5 (Ref. [59]). The final simplified risk score model presented a good discrimination performance (area under the ROC curve (AUROC) 0.808). Observed and predicted in-hospital mortality rates increased from 0% to 60% and from 1% to 65%, respectively, as the score increased from 0 up to ≥9 points. Notably, the TRI Score’s predictive accuracy surpassed that of logistic EuroSCORE and EuroSCORE II (AUROC 0.668 and 0.629, respectively) [59]. Apart from its value in predicting surgical risk, given the rapid development of TTVI, the TRI-SCORE could also serve as a valuable tool for selecting patients who may benefit from surgery or TTVI [60].

## 12. TVARC Consensus 

The TVARC consensus [11], besides the proposal STR subclassification presented above, provided important outcome definitions that could be useful to standardize TR trials, leading to more homogenous reports, accurate adjudication, and appropriate comparisons of clinical research studies.

According to TVARC Steering Committee [11], the timing of assessing endpoints is crucial for interpreting periprocedural, early, and later risks and benefits of TR therapy. The duration of follow-up must be sufficient to ascertain device durability, ensuring it is acceptable for the intended patient population and comparable to alternative therapies. Clinical outcomes should be reported at in-hospital, 30-day, and 1-year follow-up. Common safety endpoints might be assessed at in-hospital and 30-day, while less common safety endpoints and device failures may occur only after a longer follow-up. Imaging efficacy endpoints should be reported at post-procedure or predischarge, 30 days, and 1 year at a minimum, with yearly reporting up to 5 years in premarket studies.

In terms of endpoints, clinical trials should report both all-cause hospitalizations and cardiovascular and HF hospitalizations. Hospitalizations should also be adjudicated as valve, both native or device, and/or procedure-related. Commonly disease-specific instruments for HF patients include the Kansas City Cardiomyopathy Questionnaire (KCCQ) and the Minnesota Living with HF Questionnaire. Objective performance measures, which are not true patient-reported outcomes, can also be used to further quantify a patient’s physical function and health status. This includes the 6-minute walk test (6MWT), with a 25-to-50-meter increase in the 6MWT being considered a clinically significant improvement for an individual patient. Safety endpoints, including device-related complications and success endpoints should also be considered. These may involve TV reintervention, bleeding, vascular, access-related, cardiac injury, conduction disturbances, complications involving cardiac implantable electronic devices, neurological events, pulmonary embolism, deep vein thrombosis, and device- and procedure-related complications. Standardizing the reporting of these outcomes is crucial for the understanding and management of TR.

## 13. TTVI Versus Surgical Approach

Even though a comprehensive discussion regarding current TTVI options and outcomes falls beyond the purview of this surgical review, there are few reports comparing TTVI with conventional surgical approaches that are worth mentioning. In this line, Wang *et al*. [61] analyzed demographic characteristics, complications, and outcomes of 92, 86, and 84 TR patients who underwent TR surgical repair (STVr) or replacement (STVR), and transcatheter repair (TTVr), respectively, using real-world data from the National Inpatient Sample (NIS) database. The study found that TTVr patients were significantly older than STVr (65.03 years in STVr, 66.3 years in STVR, 71.09 years in TTVr, *p* < 0.05). Patients who received STVr or STVR presented a higher mortality rate (8.7% and 3.5%, respectively) compared to TTVr (1.2%), and were more likely to experience perioperative complications, including third-degree atrioventricular block, respiratory failure, respiratory complications, and acute kidney injury. Moreover, costs of care (USD$ 37,995 ± 356,008.523 STVr *vs.* USD$ 198,397 ± 188,943.082 TTVr, *p* < 0.05; USD$ 470,948 ± 614,177.568 STVR *vs.* USD$ 198,397 ± 188,943.082 TTVr, *p* < 0.05) and hospital lengths-of-stay (15.4 ± 15.19 STVr *vs.* 9.6 ± 10.21 days TTVr, *p* = 0.267; 24.7 ± 28.81 STVR *vs.* 9.6 ± 10.21 days TTVr, *p* < 0.05) were higher for STVr or STVR than for TTVr [61].

Last but not least, a retrospective observational multicentre study by Wilde *et al*. [62] showed that, despite a trend toward lower 30-day mortality with the tricuspid transcatheter edge-to-edge repair (T-TTER) (2.8% *vs.* 10.7%, *p* = 0.07), MIC-TVS led to a significantly more efficient TR reduction (*p* < 0.001), with a similar overall 1-year survival (80.4% *vs.* 78.6%, *p* = 0.67). When stratified by TRI-SCORE, 1-year survival was much better in patients at lower scores (TEER: 89.7% in TRI-SCORE <6 *vs.*67.6% in TRI-SCORE ≥6 points, *p* < 0.01; MIC: 90.0% in TRI-SCORE <6 *vs.* 50.0% in TRI-SCORE ≥6 points, *p* < 0.01) [62].

## 14. Conclusions

Despite the growing recognition that TR has received due to its prognostic role and the emergence of new interventions, TR management is still neglected. Clinical factors such as advanced stage of the disease, presence of multiple comorbidities, and high surgical risk contribute to the suboptimal outcomes associated with TR surgical interventions. These factors, along with the anatomical challenges inherent to TV, must be contemplated not only to determine the optimal timing for intervention but also to choose the most suitable surgical technique (Table 3). Therefore, addressing the neglected aspects of TR management, especially in light of its prognostic role, requires a comprehensive understanding of both clinical factors and anatomical intricacies. By doing so, we can enhance the effectiveness of interventions and improve patient outcomes.

**Table 3.** **Challenges for the tricuspid valve intervention**.

TV surgical candidates		
Clinical and epidemiological factors		
	Age	Old patients
	Comorbidities	High frequent
	Surgical risk	High surgical risk
	Preferable surgical technique	Repair
	Multiple valve disease	Frequently associated with left-sided valve disease
	Entities	PTR and STR (predominant cause)
Anatomical factors		
	Components of the valve	Tricuspid valve, RA, RV, subvalvular apparatus
	Configuration of the valve	Asymmetrical – 3 leaflets
	Morphology of the annulus	3D saddle-shaped annulus
	Dimensions of the annulus	Large annulus dimension
	Calcification	Less frequent
	Structures in proximity	Right coronary artery, coronary sinus, conduction system

PTR, primary tricuspid regurgitation; RA, right atrial; RV, right ventricle; STR, secondary tricuspid regurgitation; TV, tricuspid valve.

## Abbreviations

AF, atrial fibrillation; A-STR, atrial secondary tricuspid regurgitation; CI, confidence interval; CTEPH, chronic thromboembolic pulmonary hypertension; FAC, fractional area change; HF, heart failure; HFpEF, heart failure with preserved ejection fraction; HFrEF, heart failure with reduced ejection fraction; HR, hazard ratio; KCCQ, Kansas City Cardiomyopathy Questionnaire; LTR, lead-associated tricuspid regurgitation; LTR-A, lead-associated tricuspid regurgitation type A; LTR-B, lead-associated tricuspid regurgitation type B; LV, left ventricle; LVEF, left ventricle ejection fraction; MIC-TVS, minimally invasive tricuspid valve surgery; MR, mitral regurgitation; MV, mitral valve; OR, odds ratio; PAH, pulmonary arterial hypertension; PASP, pulmonary artery systolic pressure; PH, pulmonary hypertension; PTR, primary tricuspid regurgitation; RA, right atrial; RCT, randomized clinical trials; RHD, rheumatic heart disease; RR, relative risk; RV, right ventricle; STS, Society of Thoracic Surgeons; STR, secondary tricuspid regurgitation; 6MWT, 6-minute walk test; STVr, surgical tricuspid valve repair; STVR, surgical tricuspid valve replacement; TA, tricuspid annular; TAPSE, tricuspid annular plane systolic excursion; TR, tricuspid regurgitation; TTVI, transcatheter tricuspid valve intervention; T-TTER, transcatheter edge-to-edge repair; TTVr, transcatheter tricuspid valve repair; TV, tricuspid valve; TVARC, Tricuspid Valve Academic Research Consortium; 3D, 3-dimensional; V-STR, ventricular secondary tricuspid regurgitation.
